# Antibody and lectin target podoplanin to inhibit oral squamous carcinoma cell migration and viability by distinct mechanisms

**DOI:** 10.18632/oncotarget.3515

**Published:** 2015-03-10

**Authors:** Jhon A. Ochoa-Alvarez, Harini Krishnan, John G. Pastorino, Evan Nevel, David Kephart, Joseph J. Lee, Edward P. Retzbach, Yongquan Shen, Mahnaz Fatahzadeh, Soly Baredes, Evelyne Kalyoussef, Masaru Honma, Martin E. Adelson, Mika K. Kaneko, Yukinari Kato, Mary Ann Young, Lisa Deluca-Rapone, Alan J. Shienbaum, Kingsley Yin, Lasse D. Jensen, Gary S. Goldberg

**Affiliations:** ^1^ Departments of Molecular Biology, Cell Biology, and Pathology, School of Osteopathic Medicine, Rowan University, Stratford, NJ, USA; ^2^ Department of Diagnostic Sciences, Rutgers School of Dental Medicine, Newark, NJ, USA; ^3^ Department of Otolaryngology - Head and Neck Surgery, Rutgers New Jersey Medical School, Newark, NJ, USA; ^4^ Department of Dermatology, Asahikawa Medical University, Midorigaoka-Higashi, Asahikawa, Japan; ^5^ Medical Diagnostic Laboratories, Hamilton, NJ, USA; ^6^ Department of Regional Innovation, Tohoku University Graduate School of Medicine, Seiryo-machi, Aoba-ku, Sendai, Miyagi, Japan; ^7^ Department of Medical and Health Sciences, Linköping University, Lasarettsgatan, Ingång, Linköping, Sweden

**Keywords:** podoplanin, cancer, cell migration, receptor, lectin

## Abstract

Podoplanin (PDPN) is a unique transmembrane receptor that promotes tumor cell motility. Indeed, PDPN may serve as a chemotherapeutic target for primary and metastatic cancer cells, particularly oral squamous cell carcinoma (OSCC) cells that cause most oral cancers. Here, we studied how a monoclonal antibody (NZ-1) and lectin (MASL) that target PDPN affect human OSCC cell motility and viability. Both reagents inhibited the migration of PDPN expressing OSCC cells at nanomolar concentrations before inhibiting cell viability at micromolar concentrations. In addition, both reagents induced mitochondrial membrane permeability transition to kill OSCC cells that express PDPN by caspase independent nonapoptotic necrosis. Furthermore, MASL displayed a surprisingly robust ability to target PDPN on OSCC cells within minutes of exposure, and significantly inhibited human OSCC dissemination in zebrafish embryos. Moreover, we report that human OSCC cells formed tumors that expressed PDPN in mice, and induced PDPN expression in infiltrating host murine cancer associated fibroblasts. Taken together, these data suggest that antibodies and lectins may be utilized to combat OSCC and other cancers that express PDPN.

## INTRODUCTION

Approximately 300,000 new cases of oral cancer are diagnosed each year, causing over 120,000 deaths worldwide [1, 2]. Greater than 90% of these cancers are oral squamous cell carcinomas (OSCC) that proceed from hyperplasia to dysplasia, carcinoma in situ, and invasive carcinoma [3, 4]. Patients with early (stage I or II) OSCC are generally treated with surgery and radiation therapy, yielding 5-year survival rates between 70% and 95% [5, 6]. However, patients with more advanced OSCC (stage III or IV) have much lower 5 year survival rates, ranging from 26% to 53% [5]. Cancer recurrence is found in up to 76% of patients treated with surgery and radiation, and many of these metastasize to distant sites [7, 8]. In addition, these surgeries and radiation treatments can be disfiguring and cause acute patient discomfort (e.g. oral mucositis), as well as permanent sequelae [6, 9].

Oral cancer does not respond well to standard chemotherapeutic agents. Taxanes, anthracyclines, platinums, and antimetabolites have been used as adjuvant oral cancer therapy for several decades [9]. For example, methotrexate, cisplatin, carboplatin, 5-fluorouracil, paclitaxel, and docetaxel are commonly used to treat advanced OSCC [7]. In general, these agents have shown significant toxicity and have had little effect on outcomes [10, 11]. Collateral damage to dividing cells can cause mucositis, renal dysfunction, neurotoxicity, and haemotologic toxicities that can be debilitating and even deadly [10, 12]. In fact, over 40 years of work and clinical trials with cytotoxic chemotherapy agents have not considerably increased survival rates or quality of life for oral cancer patients [7, 11]. New treatments are clearly needed to improve outcomes in this patient population.

Targeting specific extracellular receptors can lead to successful cancer therapies. These targeted therapies include tyrosine kinase blockers that inhibit the activities of EGFR receptors (e.g. cetuximab, lapatinib) [13, 14], as well as monoclonal antibodies that target the HER2/NEU/ERB2 receptor (e.g. trastuzumab) or VEGFR2/KDR ligands (e.g. bevacizumab) [15, 16]. Indeed, cetuximab has shown promising results in clinical trials involving OSCC [17, 18]. Undoubtedly, extracellular receptors are valid targets for the treatment of human cancer.

OSCC cells present podoplanin (PDPN) as a functionally relevant biomarker and potential chemotherapeutic target. OSCC and premalignant lesions often exhibit polymorphisms in cyclin D1, and inactivation of tumor suppressors including p53, p16 and p14 [19-21]. Increased expression of tumor promoters including TIMPs, c-myc, cyclin D1, TGF-α, EGFR, and PDPN are also often seen in these lesions [19, 22-24]. Taken together, reports indicate that PDPN expression is notably increased in over 30% of pre-malignant oral lesions and in over 60% of oral cancers. Moreover, PDPN expression correlates with clinicopathological factors. About 50% of T1 and T2 primary tumors display elevated PDPN expression, and this number increases to about 75% for those at stages T3 and T4. In addition, over 70% of primary OSCC tumors with cervical lymph node metastases express elevated levels of PDPN [5, 25-28]. Clinical studies also indicate that 5-year overall survival (OS) rates continuously decrease from 93% for patients with weak podoplanin expression, to 47% for patients with moderate expression, to 23% for patients with high levels of podoplanin expression [5, 29]. OSCC lethality also correlates with PDPN expression, with undetectable, weak, moderate, and high PDPN expression resulting in 100%, 93%, 70%, and 37% 5-year disease-specific survival (DSS) rates, respectively [29]. PDPN promotes OSCC cell motility [30, 31] to drive tumor invasion and metastasis that cause most oral cancer deaths [7, 32, 33].

PDPN is a transmembrane mucin-like protein that augments tumor cell invasion. PDPN expression is induced by tumor promoters including TPA, RAS, and Src [34-36]. The Src tyrosine kinase utilizes the focal adhesion adaptor protein Cas/BCAR to induce PDPN expression in order to promote tumor cell motility [34]. PDPN regulates the activities of Rho, ezrin, and other proteins linked to the actin cytoskeleton to mediate filopodia formation, cell motility, invasion, and metastasis [37, 38]. Indeed, PDPN expression enhances the motility and invasion of several transformed cell types including mammary carcinoma [37, 39], glioma [40], and OSCC [30, 31]. PDPN is also located on lymphatic endothelial cells and cancer associated fibroblasts which can augment tumor invasion and metastasis [41, 42].

PDPN is found at the invasive front of many tumors, which is consistent with its role in promoting invasion [37, 39]. As discussed above, PDPN expression is strongly induced in most oral cancers [4, 5, 27, 28]. The bulk of the PDPN protein, about 150 amino acids, lies outside of the cell and could serve as an ideal target to combat cancer growth and progression [37, 43]. For example, antibodies against PDPN can inhibit the growth and metastasis of tumor cells that express PDPN in mice [44-47].

While antibodies may offer significant targeting specificity, they cannot be administered orally, and may not possess intrinsic pharmaceutical effects on tumor cell growth and migration seen with orally available lectins [48-50]. The extracellular domain of PDPN is *O*-glycosylated with sialic acid α2,3 linked to galactose [37]. PDPN is activated by endogenous lectins that bind to these extracellular carbohydrate moieties [51, 52] to induce tumor cell motility and metastasis [39, 53, 54]. Thus, blocking this interaction between PDPN and its ligands should inhibit malignant progression. For instance, compounds blocking the action of galectins, which activate mucin receptors, can inhibit tumor cell metastasis [55, 56]. Although some lectins may nonspecifically bind to many glycoproteins, *Maackia amurensis* seed lectin (MASL) can precisely target specific glycoproteins expressed by human cells [57, 58]. In fact, MASL, which has a high affinity for *O*-linked carbohydrate chains containing sialic acid [59, 60], targets PDPN in order to inhibit tumor cell growth and motility at nanomolar concentrations [61].

Here, we compare the effects of anti-PDPN antibody and MASL on OSCC cell motility and growth. These results indicate that both reagents affect OSCC cells at similar concentrations and by comparable mechanisms. However, MASL presents very efficient targeting dynamics and an advantage of oral administration.

## RESULTS

### PDPN expression correlates with OSCC cell motility

**Figure 1:**
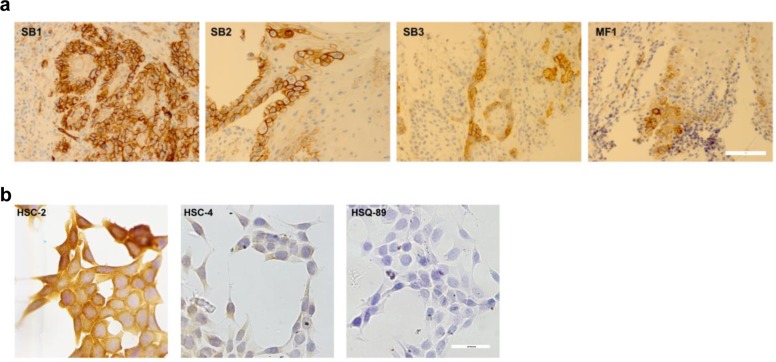
PDPN expression in OSCC cells PDPN expression in OSCC cells was examined by immunohistochemistry. (a) Specimens from oral cancer patients (bar = 40 microns). (b) Cultured OSCC cells (bar = 100 microns).

As described above, PDPN is a functionally relevant biomarker and potential chemotherapeutic target expressed by malignant OSCC cells. Immunohistochemistry found PDPN expression in OSCC specimens from oral cancer patients, as well as from a patient with leukoplakia as shown in Figure 1a and Table 1. Immunohistochemistry also detected PDPN expression in cultured OSCC cells as shown in Figure 1b and Table 2.

**Table 1:** Patient samples. Diagnosis, lesion sites, patient sex, age, PDPN expression, and HPV status are shown

Sample	Diagnosis	Site	Sex	Age	PDPN	HPV
SB1	OSCC	tongue	male	61	+++	none
SB2	OSCC	tongue	male	62	+++	none
SB3	OSCC	mouth floor	male	57	++	none
MF1	Leukoplakia	tongue	male	47	++	none

**Table 2:** OSCC cell lines. Diagnosis, lesion sites, patient sex, age, PDPN expression, and HPV status are shown

Cells	Diagnosis	Site	Sex	Age	PDPN	HPV
HSC-2	OSCC	mouth floor	male	69	+++	none
HSC-4	OSCC	tongue	male	64	++	none
HSQ-89	OSCC	maxilla	male	74	+	none

**Figure 2:**
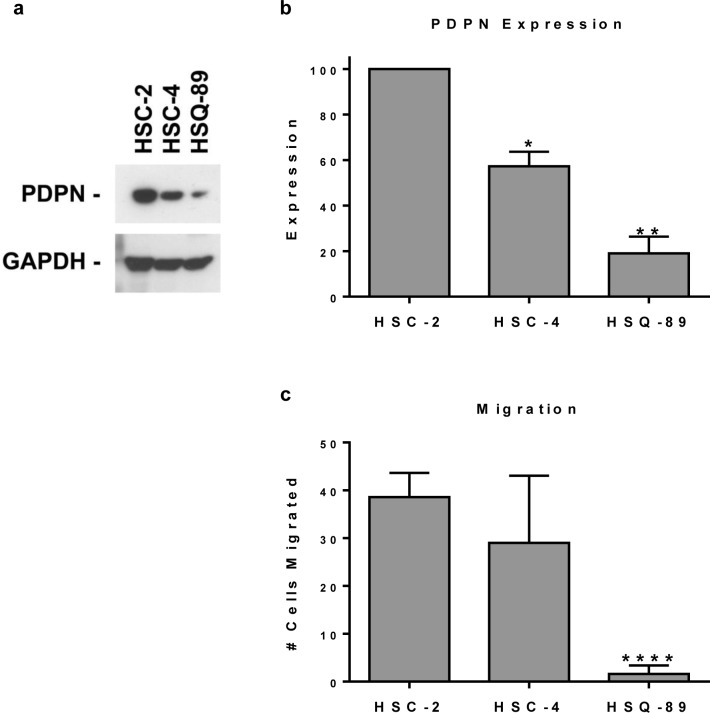
Pdpn expression correlates with OSCC cell motility (a) PDPN and GAPDH were detected by Western blotting of protein (20 μg per lane) from HSC-2, HSC-4, and HSQ-89 OSCC cells. (b) PDPN expression was quantitated by image densitometry and shown as mean+SEM (n=2). (c) Cell migration was evaluated by wound healing and quantitated as the number of cells that migrated into a 200 × 300 micron area in the center of the wound at 18 hours (mean+SEM, n=5). Single, double, and quadruple asterisks indicate p<0.05, p<0.01, and p<0.0001 compared to HSC-2 cells, respectively.

Since PDPN can promote cell migration, we sought to evaluate the relationship between its expression with OSCC cell motility. PDPN expression and cell migration were examined in a panel of cell lines generated from oral cancer patients presented in Table 2. Western blot analysis found that these HSC-2, HSC-4, and HSQ-89 cells all expressed PDPN, but at decreasing levels, respectively (Figure 2a and 2b). These data are consistent with immunohistochemistry results shown in Figure 1b and Table 2. Moreover, as shown in Figure 2c, PDPN expression levels correlated with the ability of these cells to migrate as measured by wound healing assays. These data are consistent with reports indicating that PDPN expression can increase cell migration.

### Agents that target PDPN inhibit OSCC cell motility and growth in a PDPN dependent manner

**Figure 3:**
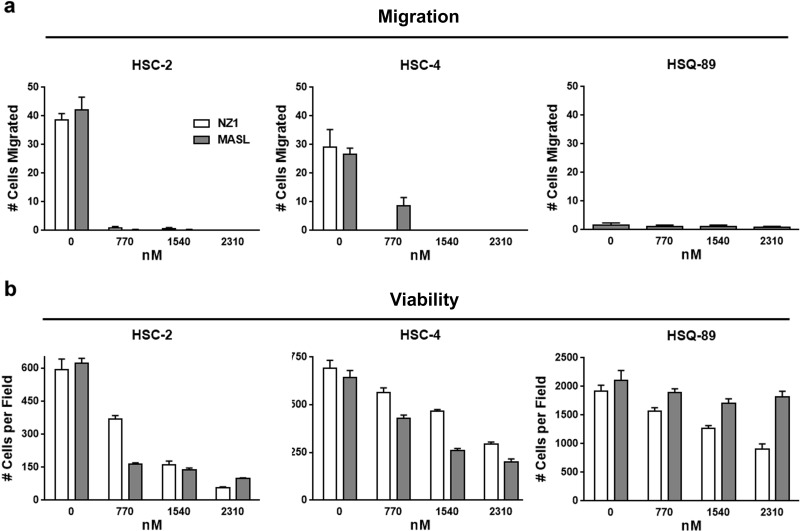
Reagents that target PDPN can decrease OSCC cell migration and viability (a) Wound healing migration assays were performed on confluent OSCC monolayers treated with concentrations of NZ-1 or MASL as indicated. Data are shown as the number of cells that migrated into a 200 × 300 micron area along the center of the wound in 18 hours (mean+SEM, n=5). (b) NZ-1 and MASL toxicity was evaluated by Trypan blue staining of cells treated with NZ-1 or MASL for 24 hours and quantitated as the number of living cells in a 3 mm^2^ field (mean+SEM, n=5).

Previous studies indicate that antibodies and lectins can be used to target PDPN in order to inhibit tumor cell migration and growth. These reagents are exemplified by NZ-1 antibody and MASL lectin [45, 61-64]. We utilized HSC-2, HSC-4, and HSQ-89 cells to evaluate the effects of NZ-1 and MASL on cell migration. As shown in Figure 3a and Supplemental Figure 1, MASL and NZ-1 both inhibited OSCC cell migration at nanomolar concentrations. Migration of HSC-2 cells, which expressed the highest levels of PDPN, was effectively inhibited by 770 nM NZ-1 and MASL. HSC-4 cell migration was effectively inhibited by 770 nM NZ-1, but required 1540 nM MASL to achieve similar results. Neither MASL nor NZ-1 showed significant effects on HSQ-89 cells, which exhibited only nominal migration and PDPN expression levels. These data indicate that NZ-1 and MASL both inhibit cell migration in a manner that correlates with PDPN expression.

In addition to inhibiting migration, MASL and NZ-1 both suppressed OSCC cell growth as shown in Figure 3b and Supplemental Figure 2. These reagents inhibited the growth of HSC-2 cells by over 80% and HSC-4 cells by over 60% at a 2.3 μM concentration. HSQ-89 cells were less sensitive to these reagents, with 2.3 μM NZ-1 inhibiting growth by about 50%, and MASL by about 15%. Since HSQ-89 cells exhibited only nominal migration and PDPN expression levels, they may not have been as effectively targeted by these reagents. Taken together, these data indicate that NZ-1 and MASL inhibited cell migration prior to suppressing cell growth. Moreover, both reagents preferentially targeted cells that express PDPN, and MASL exhibited specificity at least as strong as the NZ-1 monoclonal antibody.

**Figure 4:**
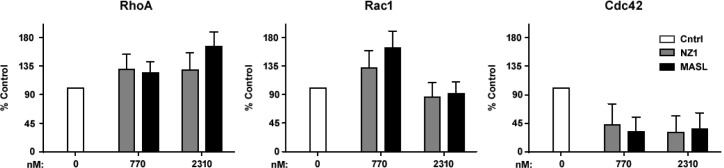
Effects of reagents that target PDPN on GTPase activity Active RhoA, Rac1, and Cdc42 GTPase was detected in HSC-2 cells treated with 0 nM, 770 nM, and 2310 nM NZ-1 or MASL as indicated. Data are shown as percent control untreated cells (mean+SEM, n=3).

RhoA and Rac1 GTPase activity has been implicated in PDPN induced cell migration [65-67]. Therefore, we sought to determine if reagents that target PDPN can decrease GTPase activity in order to inhibit OSCC cell motility and growth. As shown in Figure 4, neither NZ-1 nor MASL appeared to decrease RhoA or Rac1 GTPase activity at concentrations that inhibit OSCC cell growth or motility. Indeed, instead of decreasing RhoA and Rac1, these reagents seemed to cause a slight increase in these GTPase activities. However, in contrast to RhoA and Rac1, both NZ-1 and MASL appeared to decrease Cdc42 GTPase activity (Figure 4). These data suggest that both reagents may interfere with GTPase control of directional movement, as opposed to mechanisms directing the formation of protractions and protrusions, to inhibit cell migration, even at nontoxic concentrations (e.g. 770 nM) [68].

### NZ-1 and MASL affect OSCC cell viability without inducing apoptotic caspase activation

**Figure 5:**
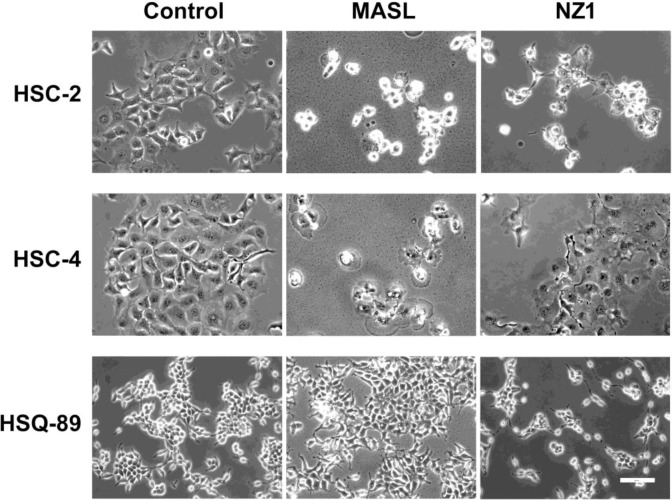
Effects of NZ-1 and MASLon OSCC cell morphology OSCC cells were treated with 2310 nM NZ-1 or MASL for 24 hours and examined by microscopy as indicated (bar = 100 microns).

Effects of NZ-1 and MASL on OSCC cell morphology suggest that these reagents caused necrotic cell death as seen in Figure 5. Although some membrane blebbing was observed, both reagents caused morphological changes indicative of necrosis in HSC-2 and HSC-4 cells. HSQ-89 cells did not exhibit notable changes in morphology, which may be expected from reduced sensitivity to NZ-1 or, particularly, MASL as shown in Figure 3.

**Figure 6:**
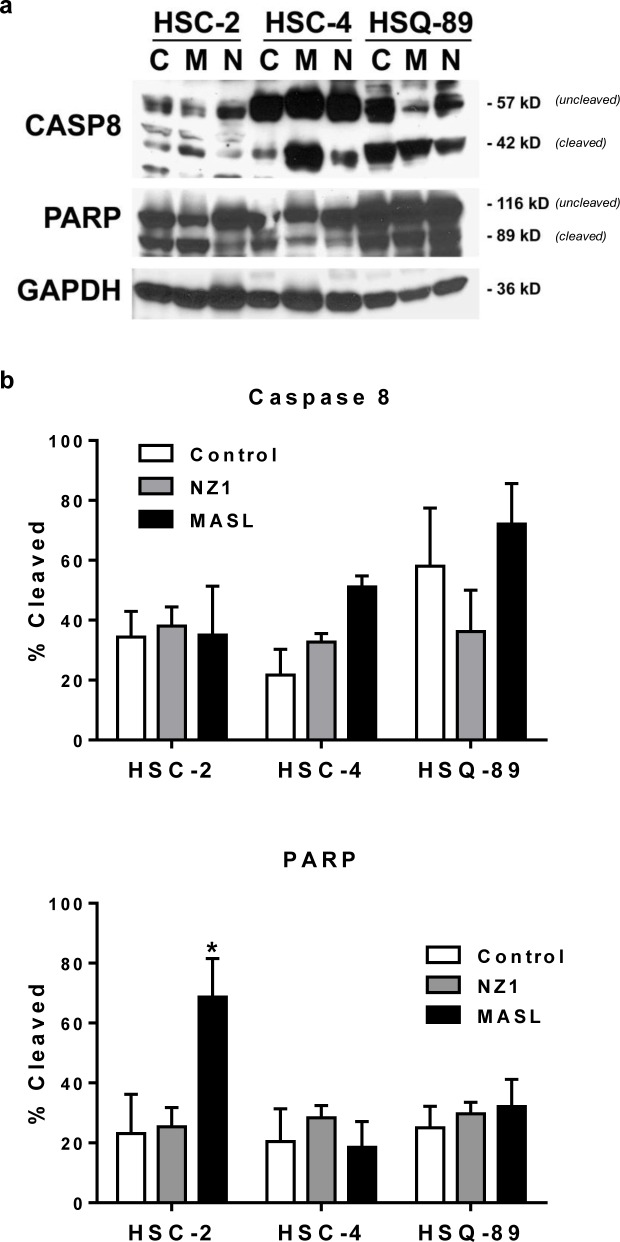
NZ-1 and MASL do not induce caspase or PARP cleavage in OSCC cells (a) PARP, Caspase 8, and GAPDH were examined by Western blotting of protein from OSCC cells treated for 24 hours with 0 nM (control) or 2310 nM NZ-1 or MASL as indicated. (b) Signal was quantitated by image densitometry and shown as the percent of cleaved PARP and Caspase 8 compared to total PARP and Caspase 8, respectively (mean+SEM, n=3).

Effects on morphology suggest that NZ-1 and MASL utilize similar mechanisms to inhibit cell growth, but that cells can react differently to these effects. For example, both compounds induced some membrane blebbing of HSC-2 cells, but not HSC-4 cells. Effects of NZ-1 and MASL on caspase 8 and PARP cleavage were evaluated to further elucidate mechanisms behind their cytotoxicity. As shown in Figure 6, NZ-1 and MASL treated cells did not display significant levels of caspase 8 cleavage in response to these compounds. While some PARP cleavage was seen in HSC-2 cells treated with MASL, this was not found in NZ-1 treated HSC-2 cells or in the other cell lines treated with either reagent (Figure 6). These data suggest that caspase activation was not essential for NZ-1 or MASL toxicity.

**Figure 7:**
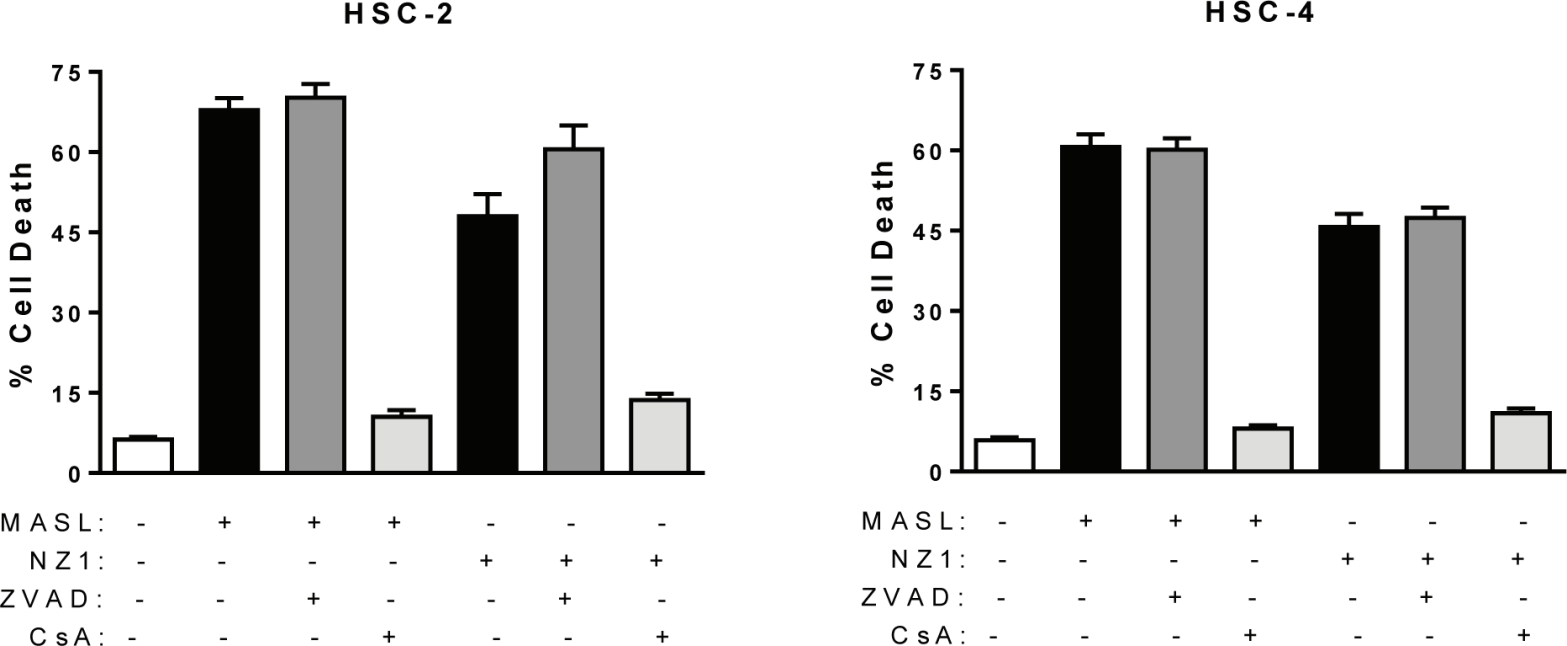
Mitochondrial membrane permeability transition inhibition protects cells from NZ-1 and MASL toxicity, while caspase inhibition does not The effects of the pan-caspase blocker Z-VAD-FMK and the mitochondrial membrane permeability transition blocker cyclosporin A were examined on HSC-2 and HSC-4 cells treated with 2310 nM NZ-1 or MASL as indicated. Data are shown as percent of cells killed (mean+SEM, n=6).

The pan-caspase blocker Z-VAD-FMK and the mitochondrial membrane permeability transition blocker cyclosporin A were used to further investigate mechanisms underlying MASL and NZ-1 toxicity in HSC-2 and HSC-4 cells. As shown in Figure 7, Z-VAD-FMK did not protect either cell line from MASL or NZ-1 toxicity. In contrast, cyclosporin A decreased toxicity of NZ-1 and MASL by several fold in both cell lines (p<0.0004 by t-test). Taken together, these data indicate that NZ-1 and MASL induce mitochondrial membrane permeability transition to kill OSCC cells by caspase independent nonapoptotic necrosis.

### NZ-1 and MASL display different OSCC cell binding dynamics

**Figure 8:**
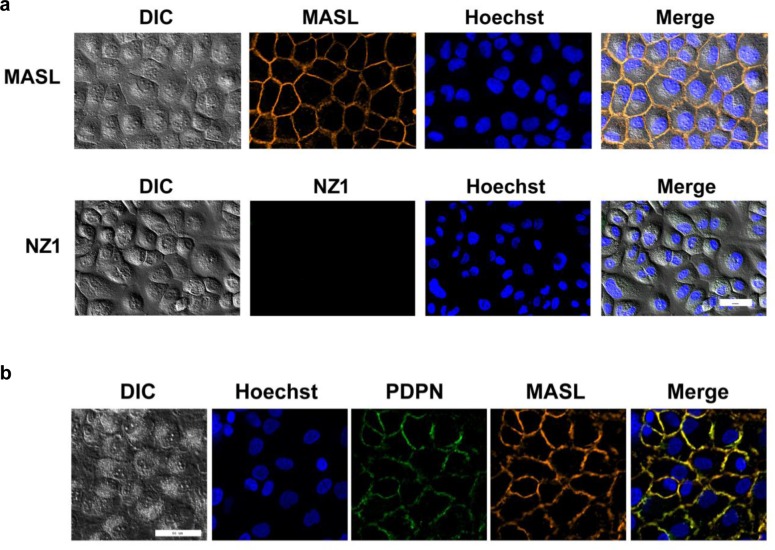
NZ-1 and MASL exhibit different OSCC cell binding dynamics (a) HSC-2 cells were incubated with fluorescently labeled MASL or NZ-1 for 2 minutes and examined by confocal microscopy. MASL bound to cell membranes, while NZ-1 did not. (b) Colocalization of MASL and PDPN is evident by confocal microscopy. Bars = 50 microns.

As shown in Figure 8a, MASL associated with the plasma membrane after only 2 minutes of exposure on HSC-2 cells. In contrast, NZ-1 was not found to associate with these cells, even after prolonged incubation periods of several hours. Immunofluorescence analysis, shown in Figure 8b and Supplemental Figure 3, found that MASL and PDPN associated with each other on the plasma membrane of these cells. Thus, MASL targeted PDPN on OSCC cells with a surprising efficiency that was not exhibited by the NZ-1 monoclonal antibody.

### MASL inhibits tumor cell invasion in zebrafish embryos

**Figure 9:**
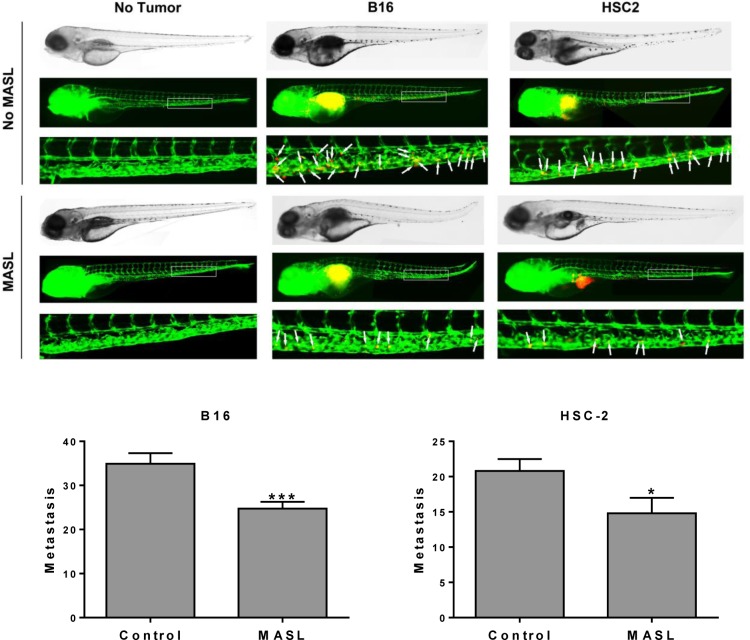
MASL inhibits melanoma and OSCC cell metastasis in zebrafish embryos (top) DiI labeled B16 melanoma and HSC-2 OSCC cells were implanted into the perivitelline cavity of zebrafish embryos grown with or without 770 nM MASL as indicated. Tumor cells (red) and blood vessels (green) were visualized after 3 days of growth. (bottom) Metastasis was quantified as the number of tumor cells that moved anterior to the anal opening (indicated by white arrows). Data are shown as mean+SEM. Single and triple asterisks indicate p<0.05 and p<0.001 (n≥20).

Zebrafish embryos provide a useful model to visualize tumor cell invasion and metastasis in living animals at the single cell level [69, 70]. We used this model to examine the effects of MASL on OSCC tumor cell dissemination. B16 melanoma cells were included in this study since previous reports indicate that MASL can inhibit their tumorigenesis in mice [61]. As shown in Figure 9, 770 nM MASL inhibited melanoma and OSCC tumor cell dissemination in this system by about 30%. This effect was significant, with p values below 0.001 and 0.05 for melanoma and OSCC cells by t-test, respectively.

## DISCUSSION

PDPN has emerged as a functionally relevant cancer biomarker and chemotherapeutic target [37, 71]. PDPN is necessary and sufficient to increase tumor cell migration. Oncogenic kinases and PDPN expression vectors augment cell migration [34, 72] and, conversely, agents including siRNA constructs that decrease PDPN expression inhibit cell migration [34, 61, 73]. This has been shown for a variety of cell types including nontransformed and Src transformed cells [34, 72], melanoma [61], and OSCC cells [73-75].

Indeed, PDPN signaling promotes invasion and metastasis of many types of cancer cells [71]. In particular, increased PDPN expression correlates with the aggressive potential of OSCC cells [76]. These cancer cells are remarkably resistant to currently available chemotherapy treatments [7, 11, 77]. PDPN could serve as a target for more effective treatments to improve outcomes in this patient population. Here, we evaluated how an antibody and lectin that target PDPN affect OSCC cell growth and motility.

Evaluation of cell lines in this study confirmed that PDPN expression correlates with OSCC cell motility. These findings are consistent with PDPN increasing cell migration. It has become very clear that PDPN acts as an important effector of signaling events that underlie cancer progression. Thus, PDPN may present an opportunity to interrupt oncogenic signaling cascades that induce its expression such as those initiated by a number of tumor promoters including Ras [35, 36], FGF/BMP [78], Src [34], EGF [73], and TGFβ [79, 80].

Quantitation of the effects of NZ-1 and MASL on OSCC cell growth and migration indicate that these compounds inhibit cell migration prior to initiating cytotoxicity. For example, MASL completely inhibits OSCC motility at 770 nM, but requires higher concentrations of over 2 μM to effectively inhibit cell viability. These studies also indicate that NZ-1 and MASL induce mitochondrial membrane permeability transition to kill OSCC cells by caspase independent nonapoptotic necrosis. Many cancer cells contain mutations that enable them to migrate and evade caspase mediated apoptosis [81]. Thus, reagents that target PDPN may provide a way to induce caspase independent necrosis in otherwise resistant human OSCC cells.

Although NZ-1 and MASL showed similar effects on cells, they displayed differences in cell binding dynamics. Effects of antibody binding to the PDPN receptor should be considered. For example, perhaps, antibody binding may stimulate PDPN cleavage (e.g. by calpain [82] or presenilin [83]) on the membrane of living cells. These results also suggest that cytotoxic effects of NZ-1 and MASL result from signaling events initiated at the plasma membrane since internalization of these compounds into PDPN expressing OSCC cells was not observed.

Human papillomavirus (HPV) infection is considered a risk factor for OSCC. However, OSCC HPV status is highly variable, and is associated with a variety of factors including geographic area and prevalence of other risk factors [84]. We found all cases evaluated in this study to be HPV negative (Supplemental Figure 4). In contrast to HPV, PDPN expression in these samples appears as a functionally relevant biomarker leading to OSCC motility.

**Figure 10:**
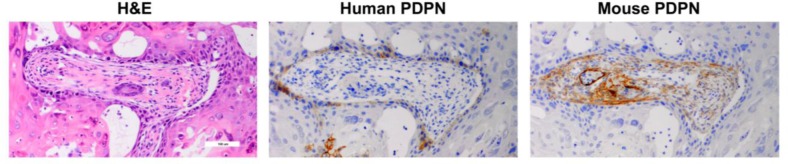
PDPN expression in human OSCC cells and infiltrating mouse fibroblasts in xenograft tumors Tumors from HSC-2 cells were examined with antisera specific for human (D2-40) and mouse (8.1.1) PDPN by immunohistochemistry as indicated.

In addition to cancer cells themselves, PDPN expression in cancer associated fibroblasts has been associated with tumor aggression and poor clinical outcomes [85]. This has been found in a variety of cancers including mammary carcinoma [86, 87], melanoma [88], lung squamous cell carcinoma [89], esophageal adenocarcinoma [90], and OSCC [91]. Interestingly, human HSC-2 cells form tumors that express PDPN in mice, and also induce PDPN expression in infiltrating mouse cancer associate fibroblasts within the stroma of the tumor as shown in Figure 10. These data are consistent with recent findings that cancer associated fibroblasts express PDPN to promote the motility and survival of neighboring tumor cells [72].

Antibodies against PDPN can be used to inhibit tumor progression [44-47]. However, *in vivo* antibody administration is challenging [48-50]. Unlike antibodies, lectins are resistant to gastrointestinal proteolysis [92-94], and can be taken orally to treat cancer [56, 93, 95]. In addition to carbohydrate modifications, lectin interactions are guided by amino acid residues of their target receptor proteins. Previous studies have shown that MASL associates with PDPN on the membrane of melanoma cells [61]. This study found that MASL can target PDPN on OSCC cells with remarkable dynamics, exceeding that of NZ-1 antibody which binds to PDPN with a dissociation constant of less than 1 nM [64, 96].

PDPN has emerged as a clear target for oral cancers and precancerous lesions [97, 98]. Previous studies demonstrate that MASL can survive digestion and enter the circulatory system to inhibit tumor progression in mammals [61]. We show here that MASL can target PDPN to inhibit OSCC cell growth and motility. However, targeting of MASL to other sialic acid modified receptors on cancer cells cannot be ruled out. Future studies should investigate this possibility. Interestingly, *Maackia amurensis* has been used for many centuries as a medicinal plant to treat ailments including cancer [99-103]. This work sheds light on potential mechanisms that may be exploited to expand our arsenal of targeted cancer treatments, particularly agents that can be administered orally.

## METHODS

### Evaluation of cell growth and migration

HSC-2, HSC-4, and HSQ-89 cells have been previously described [73], and were maintained in DMEM (Hyclone SH30021) supplemented with 25 mM HEPES (Hyclone SH30237) and FBS (Seradigm 1400-500) at 37^o^C in 5% CO_2_ and 100% humidity. Effects of reagents on cell viability were measured by plating cells at 12% confluence and growing overnight on standard 12 well tissue culture plates (Cyto One CC7682-7512), treating for 24 hours with MASL (Sentrimed) or NZ-1 (prepared as described [46, 53, 104, 105]), and counting cells after staining with Trypan blue. For wound healing migration assays, confluent cell monolayers were treated for 24 hours with MASL or NZ-1, scratched, and migration was quantitated as the number of cells that entered a 200 × 300 micron area in the center of the wound at 18 hours as previously described [61, 72].

### HPV analysis

DNA was extracted and analyzed by a proprietary HPV Type-Detect 2.0 Bio-Plex diagnostic analysis (Medical Diagnostic Laboratories, Hamilton, NJ) that was designed to detect HPV subtypes 6, 11, 16, 18, 31, 33, 35, 39, 42, 43, 44, 45, 51, 52, 56, 58, 59, 66, and 68. An internal amplification control was included for all samples to verify successful extraction and a lack of PCR inhibitors in the original specimen. Reactions also included negative template controls to calculate CT values above background as well as HPV-type specific DNA and allele specific primer extension (ASPE) positive controls to demonstrate overall assay success. Results for HPV-16 and HPV-18 were also confirmed by a proprietary multiplex real-time PCR assay (Medical Diagnostic Laboratories, Hamilton, NJ) interpreted with Rotor-Gene software (Bio-Rad, Hercules, CA).

### Immunohistochemistry

Surgical specimens were fixed in 10% formalin in PBS, paraffin embedded, sectioned (4 microns), and processed for hematoxylin/eosin staining and immunohistochemistry with 8.1.1 and D2-40 monoclonal antibodies (Dako) to detect mouse and human PDPN, respectively, as described [61, 106, 107]. OSCC cells were cultured in chamber slides (Lab-Tek 177445), fixed in 10% formalin, and processed for immunohistochemistry as described above. For mouse xenograft studies, 1 million HSC-2 cells were injected into the left flank of immunodeficient NOD scid gamma mice (Jackson Labs 005557) and allowed to form tumors which were excised and examined by immunohistochemistry. Human and mouse experimental protocols were approved by the University Institutional Review Board (study ID Pro2012001544) and Institutional Animal Care and Use Committee (APR 10579), respectively.

### Live cell imaging and immunofluorescence studies

Live cell imaging was performed on HSC-2 cells cultured on 35mm poly-D-lysine–coated glass bottom culture dishes (MatTek Corp., P35GC-1.5-14-C). Nuclei were stained with 5 μg/ml of Hoechst 33352 (Life Technologies, H1399). Cells were rinsed with PBS, incubated with 200 μg/ml MASL conjugated with red fluorescent dye (Thermo scientific Dylight 594, 53044) or 200 μg/ml NZ-1 conjugated to green fluorescent dye (Thermo scientific DyLight 488, 53024) for 2 minutes, rinsed thrice with PBS, and fed media. Images were immediately obtained on a Zeiss Axiovert microscope as described [108].

Immunofluorescence was performed on HSC-2 cells cultured on 35mm poly-D-lysine–coated glass bottom culture dishes (MatTek Corp., P35GC-1.5-14-C), fixed with 2% paraformaldehyde (PFA) in DPBS (Life Technologies, 14040-091) for 5 minutes at 4°C, rinsed with cold 1% PFA, air-dried, and rinsed again with cold DPBS. Cells were then treated with D2-40 PDPN antibody (Dako, M3619) at 1.3 μg/mL for 3 hours, washed, and then labeled with goat anti-mouse IgG conjugated to Alexa Fluor 488 (Life Technologies, A-11001) at 20 μg/mL for 1.5 hours. Cells were then rinsed with DPBS and treated with MASL conjugated with red fluorescent dye (Thermo scientific Dylight 594, 53044) at 0.4 mg/mL for 5 minutes. Nuclei were stained with Hoechst 33352 (Life Technologies, H1399). Images were obtained on a Zeiss Axiovert microscope as described [108].

### Western blotting and GTPase analysis

Western blotting was performed as described previously [61, 72]. Briefly, protein was resolved by SDS-PAGE, transferred to Immobilon-P membranes (Millipore IH1079562), and incubated with antisera specific for PDPN (NZ-1), PARP (Cell Signaling Technology 9542), Caspase 8 (Cell Signaling Technology 9746), or GAPDH (Santa Cruz Biotechnology A1978). Primary antiserum was recognized by appropriate secondary antiserum conjugated to horseradish peroxidase and detected using enhanced chemiluminescence (Thermo Scientific 32106). After blotting, membranes were stained with India ink to verify equal loading and transfer.

Active GTPase was detected in OSCC cells as described [109]. Briefly, HSC-2 cells were grown to 60-70% confluence, treated with 0 nM, 770 nM, and 2310 nM NZ-1 or MASL for 24 hours, and processed to evaluate RhoA, Rac1, and Cdc42 activity using G-LISA kits BK124-S, BK128-S, and BK127-S, respectively, according to manufacturer's instructions (Cytoskeleton, Inc.).

### Utilization of caspase and mitochondrial membrane permeability transition blockers

The Z-VAD-FMK pan-caspase blocker and cyclosporin A mitochondrial membrane permeability transition blocker were used as previously described [110-112]. Briefly, cells were grown to 40% confluence in standard culture dishes, washed and incubated for 1.5 hours with 10 μM Z-VAD-FMK (Calbiochem 219007) or 100 nM cyclosporin A (Calbiochem 239835) prior to the addition of NZ-1 or MASL, incubated for an additional 16 hours, released from the plates with trypsin, washed and resuspended in PBS with 5 mM propidium iodide for 5 minutes, pelleted, and resuspended in PBS. The percentage of viable cells was determined using a Cellometer (Nexelom, Lawrence, MA), as the ratio of the number of fluorescent cells (propidium iodide positive) to total cells.

### Zebrafish tumor cell dissemination assay

The metastatic potential of human squamous cell carcinoma (HSC-2) or melanoma (B16) cells were tested by zebrafish tumor xenograft cell dissemination assays as described [69, 70]. Briefly, tumor cells were labeled with DiI (Invitrogen 3899), suspended at 100 million cells/mL, and injected into 2 day old transgenic fli1a:EGFP embryos (ZIRC, Eugene, Oregon) anesthetized with 0.04% MS-222 (Sigma E10521) at 300-500 cells/embryo. Correct implantation was verified by fluorescence microscopy (Nikon SMZ1500 with NIS Elements F software) shortly after injection, and embryos in which cells were already present in circulation were removed. Embryos were then incubated for 3 days in PTU-supplemented E3 water without methylene blue (Sigma P7629) with and without 770 nM MASL (Sentrimed). Embryos were then anesthetized and disseminated tumor cells were counted as the number of fluorescent DiI labeled cells anterior to the anal opening at 5 days post fertilization. Three independent experiments were done with similar results.

### Statistical analyses

Statistical analyses were performed with GraphPad Prism version 6.

## SUPPLEMENTARY MATERIALS AND FIGURES


