# Supplementary material for: Antibody and lectin target podoplanin to inhibit oral squamous carcinoma cell migration and viability by distinct mechanisms
Source: Oncotarget. 2015 Mar 10;6(11):9045–60. doi: 10.18632/oncotarget.3515 (PMC4496201; doi:10.18632/oncotarget.3515)
Supplement: Supplementary file 1 [file oncotarget-06-9045-s001.pdf]

Antibody and lectin target podoplanin to inhibit oral squamous carcinoma cell migration and viability by distinct mechanisms

Supplementary Material

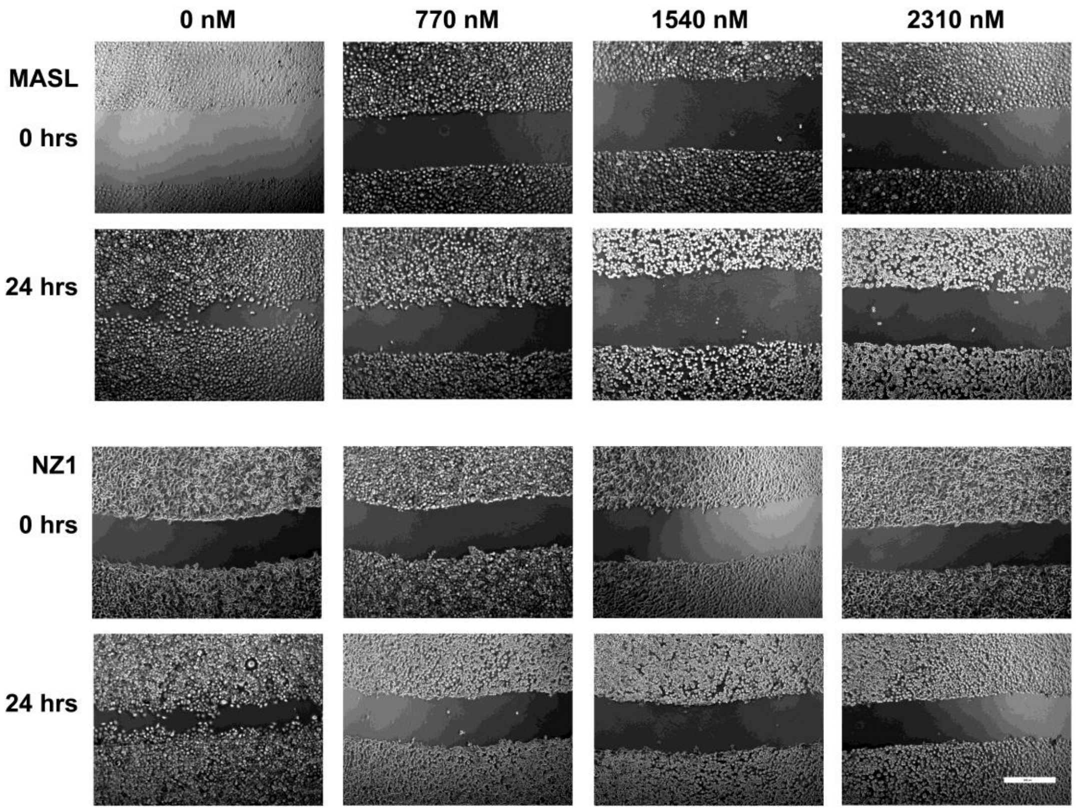

Supplemental Figure 1: Effects of NZ-1 and MASL on cell migration.

Wound healing migration assays were performed on confluent OSCC monolayers treated with concentrations of NZ-1 or MASL as indicated (bar = 500 microns).

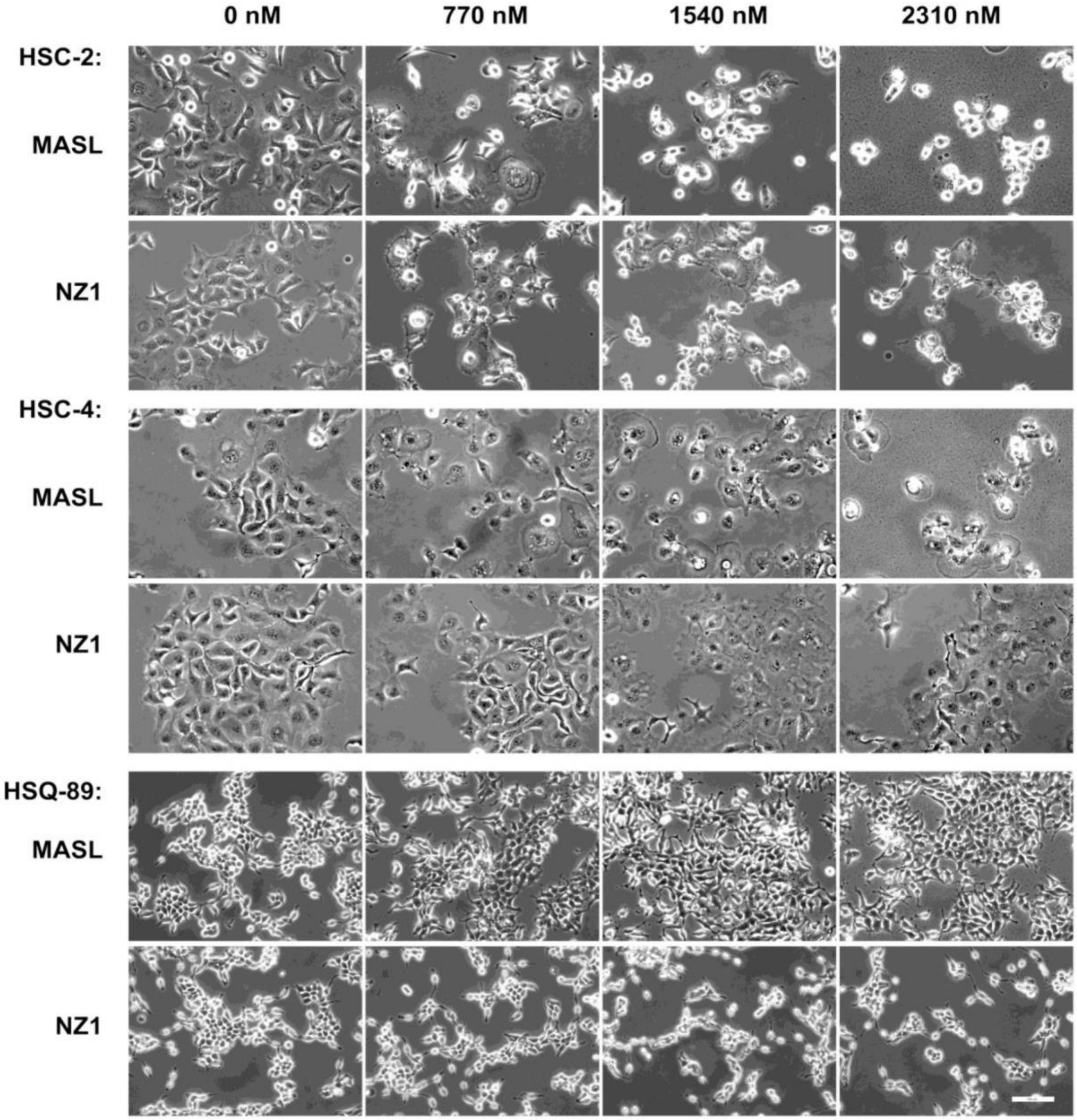

Supplemental Figure 2: Effects of NZ-1 and MASL on cell growth and morphology.

Cells were treated with NZ-1 and MASL for 24 hours as indicated (bar = 500 microns).

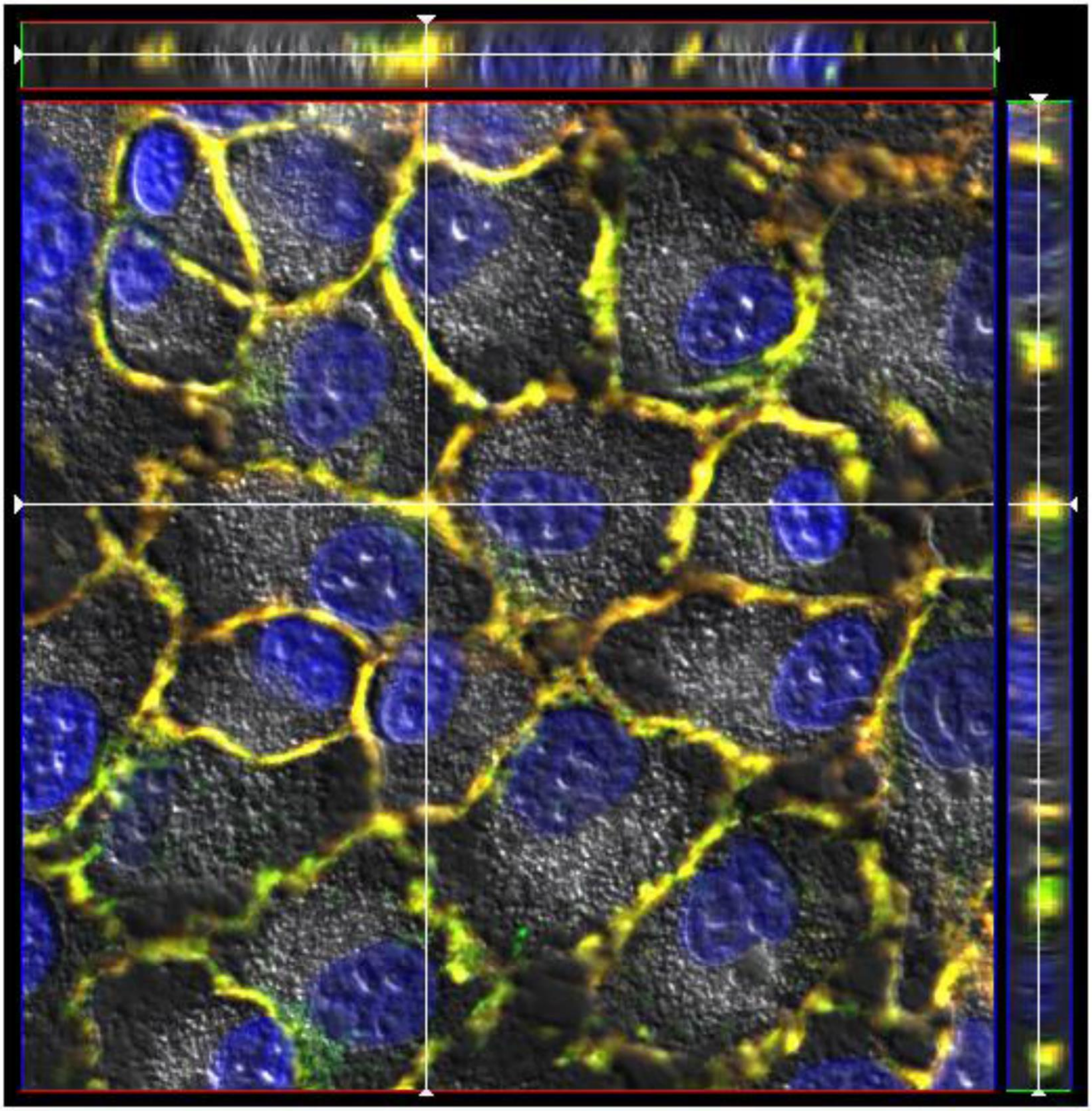

Supplemental Figure 3: Association of MASL and PDPN on OSCC cell membrane.

HSC-2 cells were incubated with fluorescently labeled MASL for 2 minutes (red), washed, stained with monoclonal antibody against PDPN (green), labeled with Hoechst to visualize nuclei, and examined by DIC and confocal microscopy. Colocalization of MASL and PDPN (yellow) is apparent in merged image, including orthogonal views of the z-axis.

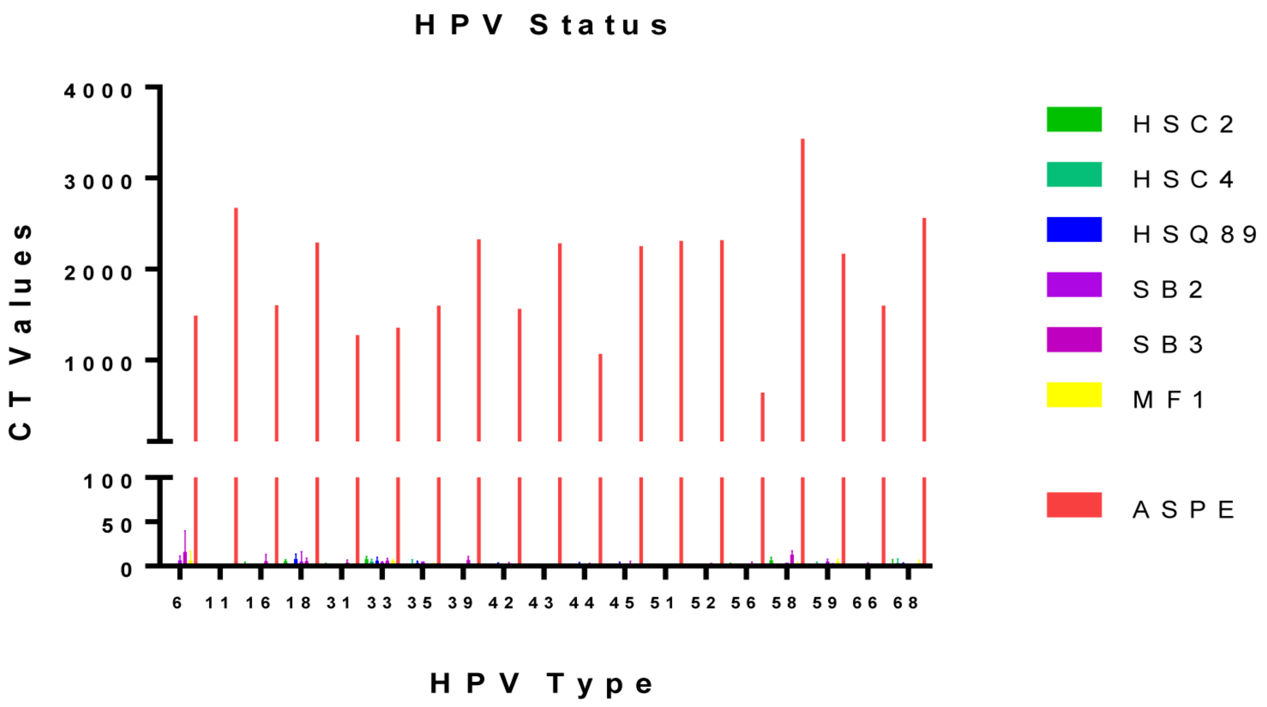

Supplemental Figure 4: HPV status of OSCC cells.

Bio-Plex PCR was used to detect DNA from 19 HPV subtypes in patient specimens as indicated. HPV was not detected in any of the samples. Data are shown as mean+SD (n=2). The results were confirmed for HPV-16 and HPV-18 by real-time multiplex PCR technology (data not shown).
